# Emotional prosodic change detection in autism Spectrum disorder: an electrophysiological investigation in children and adults

**DOI:** 10.1186/s11689-018-9246-9

**Published:** 2018-09-18

**Authors:** J. Charpentier, K. Kovarski, E. Houy-Durand, J. Malvy, A. Saby, F. Bonnet-Brilhault, M. Latinus, M. Gomot

**Affiliations:** 1 UMR1253, INSERM, Université de Tours, TOURS, France; 2 Centre Universitaire de Pédopsychiatrie, CHRU de Tours, TOURS, France

**Keywords:** Mismatch negativity (MMN), Autism spectrum disorder, Change detection, Emotion, Prosody, Children, Adults, EEG

## Abstract

**Background:**

Autism spectrum disorder (ASD) is characterized by atypical behaviors in social environments and in reaction to changing events. While this dyad of symptoms is at the core of the pathology along with atypical sensory behaviors, most studies have investigated only one dimension. A focus on the sameness dimension has shown that intolerance to change is related to an atypical pre-attentional detection of irregularity. In the present study, we addressed the same process in response to emotional change in order to evaluate the interplay between alterations of change detection and socio-emotional processing in children and adults with autism.

**Methods:**

Brain responses to neutral and emotional prosodic deviancies (mismatch negativity (MMN) and P3a, reflecting change detection and orientation of attention toward change, respectively) were recorded in children and adults with autism and in controls. Comparison of neutral and emotional conditions allowed distinguishing between general deviancy and emotional deviancy effects. Moreover, brain responses to the same neutral and emotional stimuli were recorded when they were not deviants to evaluate the sensory processing of these vocal stimuli.

**Results:**

In controls, change detection was modulated by prosody: in children, this was characterized by a lateralization of emotional MMN to the right hemisphere, and in adults, by an earlier MMN for emotional deviancy than for neutral deviancy. In ASD, an overall atypical change detection was observed with an earlier MMN and a larger P3a compared to controls suggesting an unusual pre-attentional orientation toward any changes in the auditory environment. Moreover, in children with autism, deviancy detection depicted reduced MMN amplitude. In addition in children with autism, contrary to adults with autism, no modulation of the MMN by prosody was present and sensory processing of both neutral and emotional vocal stimuli appeared atypical.

**Conclusions:**

Overall, change detection remains altered in people with autism. However, differences between children and adults with ASD evidence a trend toward normalization of vocal processing and of the automatic detection of emotion deviancy with age.

## Background

Autism spectrum disorder (ASD) is marked by persistent deficits in social communication and social interaction [1]. A key factor of this dimension is the failure to orient naturally to social stimuli [2] and in particular to display a preference for voice [3, 4] and to detect its emotional prosodic modulations [5]. Experimental investigations using event-related potentials (ERP) or functional magnetic resonance imaging (fMRI) have argued the absence of a voice-preferential brain response in ASD. In children, no voice-preferential response was evidenced [6] in relation to a larger brain response to non-vocal stimuli in subjects with ASD than in controls [7]. In adults, an absence of voice-preferential response was also originally described [8] attributed to a decreased brain activation to vocal stimuli, though this result has recently been refuted on a larger sample [9]. Overall, contradictory results emerged from the analysis of the auditory ERPs (i.e., P1, N1, P2, N2), which indexed the sensory vocal processing. For the P1 for example, several studies evidenced no group differences between controls and ASD in both children [6, 10, 11] and adults [12] while other investigations pointed to a smaller P1 amplitude [13, 14] and a longer latency [14, 15] in ASD compared to controls. Alteration of voice processing per se has thus not been fully confirmed.

The “need for sameness” described in the second dimension of the pathology has been associated with atypical brain responses to change in patients with ASD. Oddball paradigms are frequently used to elicit MMN (mismatch negativity) and eventually P3a responses indexing respectively automatic detection of change and automatic orientation of attention toward the change. In subjects with ASD, the severity of the symptomatology is positively correlated with an atypical MMN response (i.e., shorter MMN latency) [16]. Alterations of automatic detection processing were evidenced for changing stimuli without any social information [13, 16, 17]. Such pre-attentional detection process can be triggered by change, but also by emotion. Hence, alterations of this mechanism could be found for both non-social and social stimuli and could constitute a general impairment in patients with ASD, which could be related to the two dimensions of the pathology.

Most MMN studies using vocal stimuli focused on the discrimination of sound features (e.g., intensity) or speech changes. Investigations of speech change detection in ASD have shown some particularities. In preschool-age children with ASD, an absence of MMN was observed in response to speech syllable change [4]. In both school-age children and adults, change of vowel or consonant resulted in a similar amplitude between clinical groups but a delayed MMN was found in ASD compared to controls [12, 13, 18, 19]. Smaller P3a was also observed in response to speech change in children and adults with autism compared to controls [12, 18, 20]. Finally, when physical attributes (e.g., fundamental frequency, intensity,…) of speech stimuli varied, several alterations of MMN and P3a amplitude and latency were evidenced. MMN amplitude was found either larger, smaller, or similar between controls and ASD. Similarly, MMN laterality or topography were also reported as different or similar between groups [11, 12, 18, 20–22]. For P3a, amplitude was described as smaller or typical in people with ASD compared to controls while the latency appeared shorter, longer or similar [12, 18, 20, 22]. Overall, these few studies question the existence of an atypical detection of change in speech stimuli in autism, regardless of age.

Subsequent research showed interest in the evaluation of vocal prosodic change detection in people with ASD. Emotional voices are prosodic stimuli that contain great amounts of social information. Their interpretation has even been associated with social competence during childhood and adolescence [23]. Atypical prosodic production is a hallmark of autism and was linked to social awkwardness [24] and to poor communication and socialization skills of children with ASD [25]. These production issues might be related to an atypical perception of the prosody of vocal stimuli [26]. Behavioral studies conducted in children and adults with ASD predominantly reported lower performances on tasks of emotional prosody perception compared to controls [5, 26–34]. However, some other investigations showed similar performances between groups [35–39] but patients included in these studies had a good cognitive level and thus could have developed compensatory strategies to succeed at these active tasks. Thus, performances of patients at behavioral tasks could remain uninformative about potential alterations of brain processes involved in the processing of vocal emotional stimuli especially considering that a large part of the ASD population was not represented in these studies.

The few studies that have attempted to analyze sensory ERPs to emotional voices showed a reduction of amplitude in people with ASD compared to controls [40, 41]. Only four studies have addressed the automatic detection of emotional vocal change in ASD so far. In children with Asperger syndrome (AS), using a paradigm with a word uttered with either tender or commanding prosodies as standard and deviant, respectively, the emotional change detection elicited a double-peaked MMN response [40]. The early MMN component was lateralized to the right hemisphere in control (CTRL) children but not in the AS group. Moreover, the late MMN latency was shorter in the AS group compared to the CTRL group. In another study in children with ASD with low verbal skills [42], a neutral standard and three emotional deviants (scornful, sad, and commanding prosodies) were presented. Scornful MMN and P3a amplitudes were reduced in children with ASD compared to CTRL while no group differences were reported for the other emotions. In adults with AS, using the same paradigm, smaller MMN amplitude was evidenced in the right hemisphere for the scornful deviant compared to controls [43]. For the commanding deviant, MMN displayed a different topography in the AS group and a delayed latency. Here again, the sad MMN remained intact in ASD. Another study showed smaller P3a amplitude for angry condition in adults with ASD compared to controls [41]. Overall, these studies described either impaired or intact automatic detection of emotional changes depending on the population (age and diagnosis), the paradigm, and the emotion. Though comparing different emotional vocal stimuli, most of these studies did not control for acoustic features. Emotional prosody is based on first-order acoustical variations (e.g., fundamental frequency, intensity, …) known to modulate MMN [44]; subjects with ASD are particularly sensitive to the influence of these factors [16, 17], yet they have not been controlled making it difficult to assess the origin of group differences. The use of acoustically matched non-vocal sounds in Fan and Cheng study [41] showed that most of the between groups amplitude differences evidenced in response to emotional sounds were related to pure acoustic variations and can be found with stimuli without any emotional content. Moreover, none of these studies compared the detection of emotional deviancy to neutral deviancy; thus, the impact of the emotional component per se could not be determined. Finally, potential differences regarding these brain processes between children and adults with ASD are still unknown. In CTRL, prosodic deviancy triggered a right-lateralized MMN [45] that appeared to be either earlier or larger [45–51] in response to emotional than to neutral deviancy in adults while no emotion-related differences were found in children [45]. Altogether, age-related modifications of emotional change detection are still poorly investigated in both CTRL and ASD. Several essential questions regarding specific vocal emotional change detection in ASD thus remained unanswered.

Do people with autism present an atypical detection of change regardless of prosody (neutral, emotional) or do they have a specific alteration of emotional change detection? In view of the previous findings, we hypothesize that people with autism present an alteration of the detection of change regardless of prosody (neutral, emotional), together with an impairment for emotion-specific change detection. Based on the scarce literature on emotional prosody change detection, we predicted that MMN and P3a amplitudes would be reduced in ASD groups compared to CTRL. However, given the inconsistency of previous findings regarding MMN latency, no specific hypotheses could be drawn about this parameter. In addition, we also predicted an atypical response to the emotional change, characterized by a default of lateralization of the emotional MMN in children with ASD compared to CTRL children in whom emotional MMN appears right-lateralized. To address these hypotheses, a paradigm composed of stimuli with tightly controlled acoustic parameters was used to elicit brain responses to neutral and emotional deviancy. These responses were compared between groups (CTRL, ASD) and across age (children, adults) to evaluate potential age-related differences.

## Methods

### Participants

Fifteen children with ASD (7–11 years) and 16 adults with ASD (18–37 years; Table 1) were recruited through the Child Psychiatry Department and the Autism Resource Centre of Tours. An experienced team of clinicians diagnosed the participants according to DSM-5 criteria [1] and by using ADI-R and ADOS [52, 53]. Fifteen healthy children and 16 healthy adults also participated in the study as control participants (CTRL; Table 1). None of the CTRL reported any developmental difficulties in language or sensorimotor acquisition. For all participants, no disease of the central nervous system, infectious or metabolic disease, epilepsy, or abnormal audition was reported. Although most patients were not medicated, we report neuroleptic treatment for three adults and one child, anxiolytic for one adult, and methylphenidate for two children. Intellectual quotients (verbal, performance) were obtained for 30 patients with psychometric tests adapted to their cognitive level [54, 55]. An estimation of verbal and performance IQ was performed in CTRL using four subtests (vocabulary, similarities, block design, and matrix) of the age-adapted Wechsler intelligence scales. Two-tailed *t* tests were used to determine if verbal and performance IQ differed between CTRL and ASD and also between children and adults with ASD. Both verbal and performance IQ scores were significantly lower in the ASD population than in the CTRL population. Moreover, the verbal IQ was significantly reduced in children with ASD as compared to adults with ASD (Table 1).

**Table 1:** Group characteristics

	Children		Adults	
	CTRL	ASD	CTRL	ASD
Age ^a^	9.8 ± 1.4	10.0 ± 1.4	26.2 ± 6.4	26.2 ± 6.8
Sex ratio ♂/♀	12/3	13/2	14/2	14/2
Verbal IQ ^b,c,*^	118 ± 19	75 ± 29	116 ± 16	95 ± 18
Performance IQ ^b, *^	116 ± 16	81 ± 16	113 ± 12	90 ± 21
ADOS ^d^	–	13 ± 6	–	13 ± 4

^a^Mean age (years) ± SD

^b^Verbal and performance IQ measured with Wechsler intelligence scale (average on 15 adults with ASD)

^c^Significant difference between children and adults with ASD (*p* = .03)

^d^ADOS sum of social interaction and communication scores (average on 14 children with ASD)

*Significant differences between CTRL and ASD (*p* < .05) for both children and adults

Informed written consent was obtained from all adult participants or from their legal guardian and from children’s parents. Of course, the entire experiment was performed with the assent of all participants (children or adults). The protocol was approved by the Ethics Committee of the University Hospital of Tours and complied with the principles of the Declaration of Helsinki.

### Experimental design

The vowel /a/ uttered by different female speakers with either neutral or emotional prosody (anger, fear, happiness, surprise, disgust, sadness) was recorded with Adobe Audio 2.0. Stimuli were edited in order to have the same duration (400 ms) and loudness (70 dB SPL) and were validated on neurotypical samples of adults (*n* = 16; valence and emotion recognition) and children (*n* = 18; valence recognition) [45]. Selected stimuli displayed close mean fundamental frequencies (220–231 Hz).

**Fig. 1:**
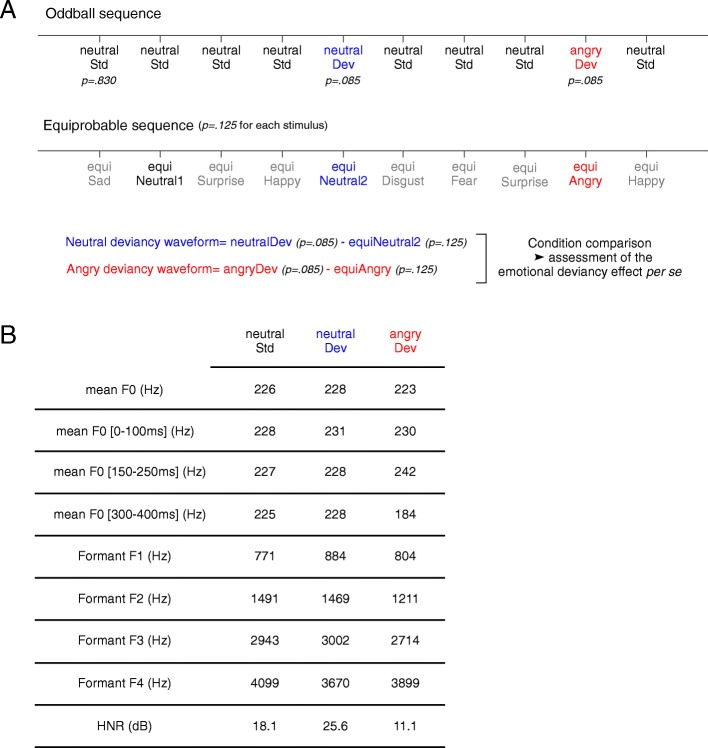
**a** Illustration of oddball and equiprobable sequences composed of neutral standard (neutralStd) and neutral and angry deviants (neutralDev and angryDev) in the oddball sequence and of equiNeutral1, equiNeutral2, equiAngry, equiSurprise, equiHappy, equiDisgust, equiFear, and equiSad in the equiprobable sequence. Black, blue, and red ink colors highlight that the three stimuli of the oddball sequence were also presented in the equiprobable sequence. **b** Acoustic properties of all stimuli of interest

During EEG recording, participants were asked to watch a silent movie without subtitles while the sounds were delivered through speakers. Automatic detection processes were studied using passive oddball and equiprobable sequences to control for both sensory processing and neuronal adaptation effects [56]. The oddball sequence comprised 1172 neutral standards (neutralStd; identity 1; probability of occurrence, *p* = .83), 120 neutral deviants (neutralDev; identity 2), and 120 angry deviants (angryDev; identity 3 and emotional deviant) (*p* = .085 each), with the constraint that two deviants were separated by a minimum of three standards. The second sequence was composed of eight different stimuli presented with an equal probability of occurrence (*p* = .125; 120 stimuli): two neutral stimuli (equiNeutral1, equiNeutral2: the neutralStd and the neutralDev from the oddball sequence) and six emotional stimuli representing the six basic emotions (equiHappy, equiSad, equiSurprise, equiDisgust, equiFear, and equiAngry, i.e., angryDev of the oddball sequence). None of the stimuli were repeated more than two times in a row in order to avoid creation of a regularity pattern. The stimulus onset asynchrony was 700 ms (total recording time: 28 min). In order to obtain the brain response to deviancy detection (Fig. 1), the neutral difference wave was obtained by subtracting the ERP elicited by equiNeutral2 from that elicited by neutralDev (neutral deviancy waveform = neutralDev − equiNeutral2; same sound in equiprobable and oddball sequences). The same subtraction (angry deviancy waveform = angryDev − equiAngry) was applied to obtain the emotional difference wave. Since stimuli in the equiprobable sequence have identical acoustic characteristics and similar probability of occurrence as the oddball deviants, the resulting difference wave more likely reflects a genuine MMN than in the oddball paradigm [57]. Moreover, the application of this subtraction process to the emotional condition allowed to control for the influence of the emotional processing which operates in both sequences, in order to isolate the effect related to emotional deviancy. Finally, the direct comparison between neutral and emotional difference waves contrasted “identity deviancy” (neutral deviancy waveform) and “identity/emotion deviancy” (angry deviancy waveform) leaving only the emotional deviancy as a differential factor between conditions, which allowed the assessment of a specific emotional deviancy effect (Fig. 1). This direct condition comparison was performed with ANOVAs, which will be detailed later in the “Statistical analysis” section.

### EEG recording and ERP measurements

The EEG was recorded from 64 active electrodes (ActiveTwo Systems Biosemi, The Netherlands) with a sampling rate of 512 Hz while eye movements were monitored using electrodes placed on left and right outer canthi and below the left eye. An electrode was placed on the nose of the subject, and data were re-referenced offline to its potential. The ELAN software package was used for the analysis of EEG-ERP [58]. The EEG signal was amplified and filtered (0.3 Hz high-pass filter). Artifacts resulting from eye movements were removed using independent component analysis (EEGlab), and movement artifacts were discarded manually. A 30 Hz low-pass filter was applied and ERP were averaged on a 800-ms time window including a 100-ms prestimulus baseline.

Artifact-free trials were averaged for each stimulus of interest (neutralDev, angryDev, equiNeutral2, equiAngry) in CTRL adults (107 ± 9, 107 ± 13, 106 ± 12, 107 ± 9), in adults with ASD (103 ± 8, 104 ± 11, 104 ± 10, 102 ± 10), in CTRL children (88 ± 14, 89 ± 14, 88 ± 15, 86 ± 14), and in children with ASD (98 ± 7, 99 ± 8, 91 ± 14, 94 ± 13). A main ANOVA performed on these data with condition (neutral, emotional) as within-subject factor and age (children, adults) and group (CTRL, ASD) as between-subject factors, revealed no condition effect. An age effect (F(1,58) = 29.16, *p* < .001, *η*_p_^2^ = .33) was reported due to a higher number of artifact-free trials in adults than in children, which is classical in developmental studies. Moreover, a group by age interaction (F(1,58) = 4.77, *p* = .033, *η*_p_^2^ = .08) was also observed because of a higher number of artifact-free trials in children with ASD than in CTRL.

In the entire manuscript, the terms “sensory/vocal processing” will refer to obligatory auditory evoked potentials (P1, N1, P2, N2) to vocal stimuli while “change/ deviance detection” will refer to results related to MMN/P3a.

One cannot exclude that a poor sensory encoding of the acoustic characteristics of each sound could influence the detection of changes between different sounds. Therefore, an analysis of sensory responses appears as an essential control to estimate the potential involvement of atypical sensory processing in MMN/ P3a results. In line with this idea, an analysis of auditory ERP was performed on P1 component, which is commonly observed in both children and adults in response to vocal stimuli. Peak amplitude and latency of P1 elicited by the equiNeutral2 and equiAngry stimuli were measured in a 50–150-ms time window in children and adults. Peak amplitudes and latencies of the MMN and P3a were measured in each subject on neutral and angry deviancy waveforms (Fig. 1). MMN was identified as the negative deflection occurring in a 120–220-ms time window for children and in a 110–210-ms time window for adults. P3a was identified as the positive deflection occurring between 240 and 340 ms and 230 and 330 ms for children and adults, respectively.

### Statistical analysis

P1 amplitude and latency to equiNeutral2 and equiAngry were analyzed with a mixed-design ANOVA performed on the electrode where the response culminates (Fz) with condition (neutral, emotional) as a within-subject factor and age (children, adults) and group (CTRL, ASD) as between-subject factors.

In order to assess differences between groups after P1, which is the only peak clearly identified in all conditions and groups, randomizations were realized for each stimulus (equiNeutral2 and equiAngry) on a 50–500-ms time window on all electrodes with a Guthrie-Buchwald correction over 25 ms [59]. Such analysis allowed determining periods of between groups’ statistical differences and constituted a good alternative for processing data, which do not display measurable components.

Two-tailed *t* tests were used to determine whether the amplitudes of evoked potentials cited below (MMN and P3a) significantly differed from zero.

A main mixed-design ANOVA analysis was performed for MMN amplitude on the electrodes where the response culminates (F3, Fz, F4, FC3, FCz, FC4, C3, Cz, C4, CP3, CPz, CP4, P3, Pz, P4) with condition, anterior-posterior (frontal, fronto-central, central, centro-parietal, parietal) and laterality (left, medial, right) as within-subject factors and age and group as between-subject factors. This selection of electrodes is consistent with previous MMN studies of emotional change detection in ASD [40–43]. MMN latency was evaluated with a mixed-design ANOVA on Cz with condition as within-subject factor and age and group as between-subject factors.

Mixed-design ANOVA was performed over Fz, FCz, and Cz for P3a amplitude with condition and anterior-posterior (frontal, fronto-central, central) as within-subject factors and age and group as between-subjects factors while a mixed-design ANOVA was realized on FCz for P3a latency.

Greenhouse-Geiser correction was applied when necessary. For significant results, the effect sizes are shown as *η*_p_^2^. Post-hoc analysis (Newman-Keuls) was performed when needed to determine the origins of interactions.

For each significant result involving the factor “Group,” correlations between electrophysiological data (i.e., MMN and P3a amplitude and latency) and verbal/performance IQ scores were calculated to estimate the potential influence of cognitive skills on the measures of evoked potentials. To assess the significance of the correlation in each group, permutations were used to generate 15,000 theoretical correlations based on random pairs of IQ score and electrophysiological measure. This operation gave a distribution of correlation slopes under the null hypothesis of an absence of correlation. Observed correlations were considered significant if they fell outside the 95% CI (confidence interval) of the theoretical distribution. *P* values were calculated by counting the number of times the random samples provided value of slopes greater than the empirical one. Confidence Intervals around the slope were estimated by bootstrapping with replacement of electrophysiological/IQ pairs within each group.

To assess differences between ASD and CTRL groups, individual participant data were permuted across groups, that is, individuals were randomly assigned to either the ASD or the CTRL group; correlations were computed between IQ scores and electrophysiological data for permuted groups. Theoretical differences between slopes of permuted groups were computed. This operation was repeated 15,000 times to generate a theoretical distribution of group differences under the null hypothesis (no ASD/CTRL difference) with a 95% CI. Empirical group differences between slopes were deemed significant if they fell outside the 95% CI. Significant threshold for these tests was adjusted for multiple comparisons with a Bonferroni correction (*p =* .0063 for eight comparisons in adults and *p =* .0056 for nine comparisons in children).

## Results

### Obligatory auditory ERPs to voice

**Fig. 2:**
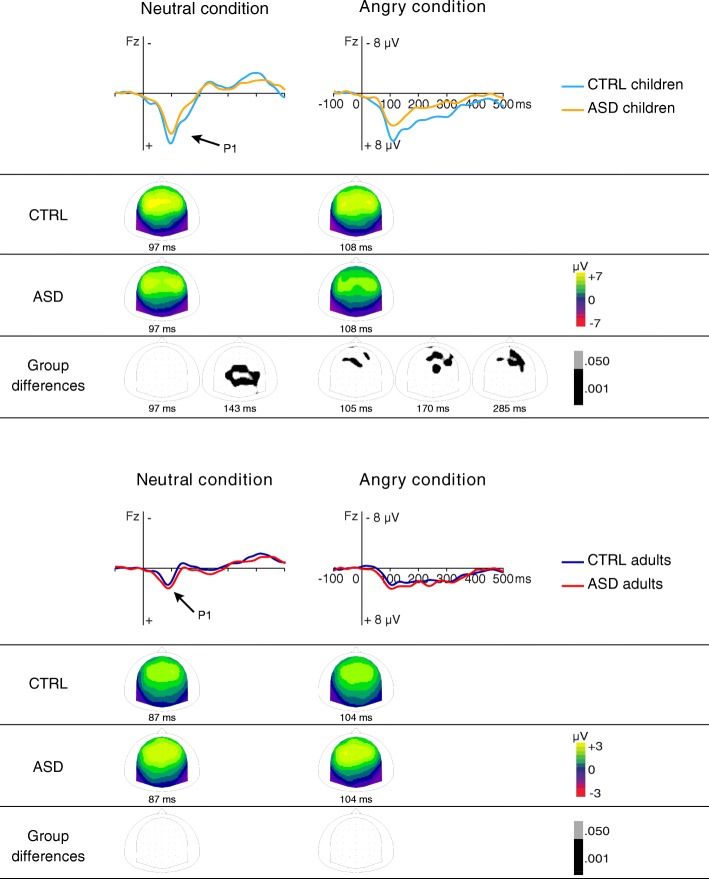
Grand average auditory brain responses to neutral (equiNeutral2) and angry (equiAngry) stimuli. Scalp distribution of the P1 response is displayed for each group along with group difference scalp distributions obtained with randomizations performed on the 50–500-ms time window (Guthrie-Buchwald time correction). Amplitude differences were observed between children groups for equiNeutral2 (123–181 ms) and for equiAngry (92–127 ms, 152–193 ms, 255–322 ms) while no differences were seen between adult groups

Larger P1 amplitude was observed in CTRL children than in children with ASD (*p* = .003) while no difference was observed between groups in adults (group by age interaction, F(1,58) = 8.61, *p* = .005, *η*_p_^2^ = .13; Fig. 2). In both groups, P1 amplitude was generally larger in children than in adults (age effect, F(1,58) = 80.17, *p* < .001, *η*_p_^2^ = .58; Fig. 2). Condition did not affect P1 amplitude.

Earlier P1 was evidenced in participants with ASD compared to CTRL participants (group effect, F(1,58) = 6.84, *p* = .011, *η*_p_^2^ = .11). A P1 latency shortening was also observed in adults compared to children in the neutral condition for both groups (*p* = .001; condition by age interaction, F(1,58) = 5.89, *p* = .018, *η*_p_^2^ = .09).

Randomizations performed over all electrodes in the 50–500-ms time window (Fig. 2) revealed amplitude differences between children groups (more positive response in CTRL than in ASD) for equiNeutral2 (123–181 ms) and for equiAngry (92–127 ms, 152–193 ms, 255–322 ms) while no amplitude differences were observed between adult groups.

### Discrimination of neutral and emotional prosodic changes

**Fig. 3:**
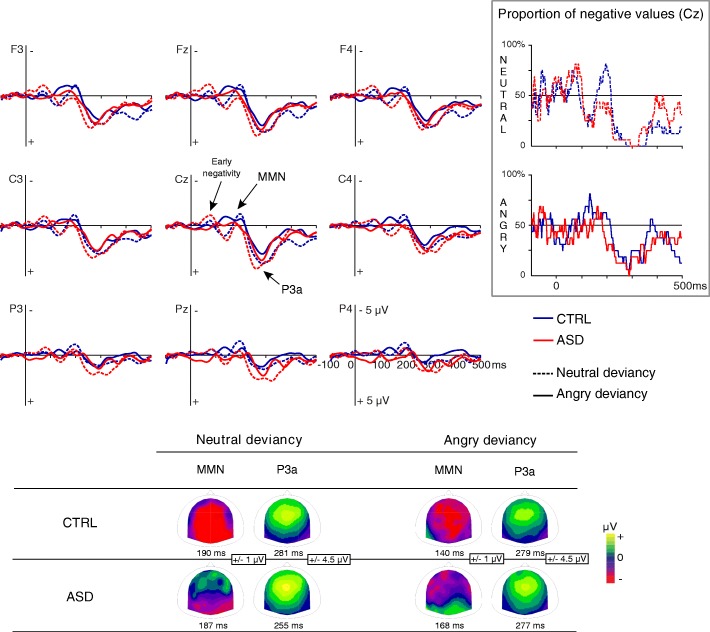
Brain responses to neutral and angry prosodic deviancies in CTRL adults and ASD. ERP of CTRL and ASD are displayed in blue and red line, respectively. Scalp distributions of MMN and P3a are displayed at the bottom of the figure. In the upper right corner, the proportion (%) of participants displaying a negative response at Cz is represented at each time point for neutral and angry deviancies in CTRL and ASD

**Fig. 4:**
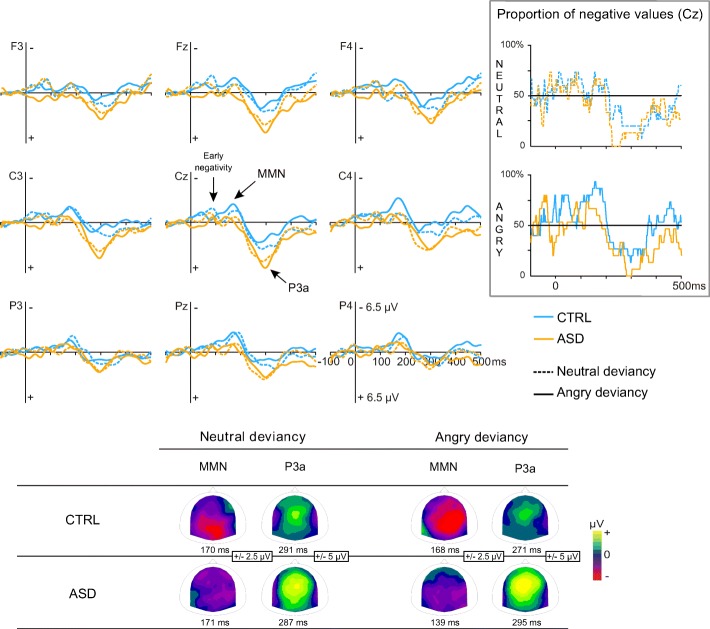
Brain responses to neutral and angry prosodic deviancies in CTRL children and ASD. ERP of CTRL and ASD are displayed in light blue and orange line, respectively. Scalp distributions of MMN and P3a are displayed at the bottom. In the upper right corner, the proportion (%) of participants displaying a negative response at Cz is represented at each time point for neutral and angry deviancies in CTRL and ASD

In children and in adults, MMN and P3a were elicited by both neutral and emotional changes (Figs. 3 and 4). An early negativity was also observed in children regardless of group and condition.

All the deflections studied in this section significantly differed from 0 (two-tailed *t* tests; *p* < .05).

#### MMN amplitude

**Fig. 5:**
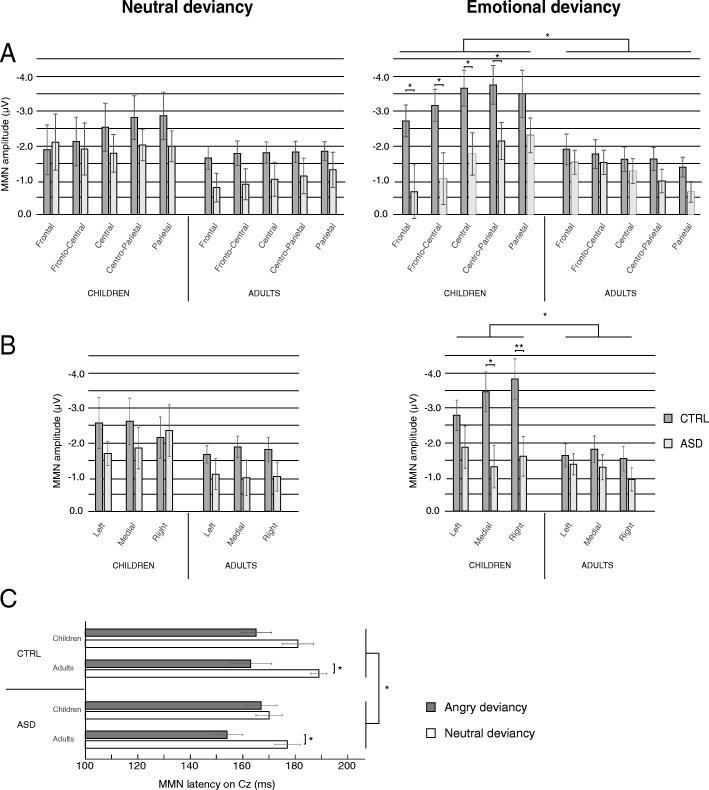
MMN amplitude (**a**, **b**) and MMN latency (**c**) for neutral and emotional deviancies. Main effects of group (CTRL/ASD) and age are reported for MMN amplitude while main effects of group and condition are shown for MMN latency. *p ≤ .05, **p ≤ .001. Barr errors represent the standard error to the mean

The main analysis performed on MMN amplitude evidenced a group effect (F(1,58) = 5.04, *p* = .029, *η*_p_^2^ = .08) with a larger amplitude in CTRL than in ASD and an age effect (F(1,58) = 5.71, *p* = .020, *η*_p_^2^ = .09) with a larger amplitude in children than in adults. This main analysis also revealed significant interactions on MMN amplitude between condition, anterior-posterior, age, and group (F(4,232) = 3.87, GG-corrected *p* = .034, *η*_p_^2^ = .06; Fig. 5a) and between condition, laterality, age, and group (F(2,116) = 3.39, *p* = .037, *η*_p_^2^ = .06; GG-corrected *p* = .054; Fig. 5b). As these interactions revealed that scalp distributions were different in children and in adults, MMN data were further analyzed separately for adults and children.

Amplitude differences were analyzed with mixed-design ANOVA performed on Fz, FCz, Cz, CPz, and Pz in adults and on Cz, CPz, and Pz in children accordingly to the MMN locations in age groups. Indeed, in both groups of children, MMN displayed posterior distribution with reduced amplitude over fronto-central site. Previous studies [60, 61] also reposted a posterior negativity in school-aged children in the MMN latency range in response to vowel or syllable deviancy. Thus, depending on the characteristics of the stimuli used to elicit the mismatch process, the MMN distribution using vocal speech stimuli in children may or may not display the classical fronto-central distribution, which guided our electrode selection for the analysis.

In adults, regardless of the group, angry MMN displayed a fronto-central topography (*p* < .005) whereas neutral MMN displayed a broader topography (condition by anterior-posterior interaction, F(4,120) = 8.56, GG-corrected *p* < .001, *η*_p_^2^ = .22; Fig. 3). No group or condition effects and no other interactions were observed on MMN amplitude. Despite this lack of significant group difference on amplitude, the observed proportion of adults with autism displaying a negative response at MMN latency appeared to differ from CTRL (Fig. 3). Hence, inter-individual variability in the ASD group might hide potential group differences as visual observation of the difference wave highlights that MMN tended to be smaller in ASD than in CTRL.

In children, MMN amplitude was significantly smaller in ASD compared to CTRL children, regardless of condition (group effect, F(1,28) = 4.40, *p* = .045, *η*_p_^2^ = .14; Fig. 4). As proportions of children displaying a negative response appeared similar between groups (Fig. 4), this result appeared genuine. No condition effect and no interaction involving the group were shown.

As no interaction between group and electrode were shown, correlations between IQ measures and MMN amplitude were performed on the mean peak amplitude over the centro-parietal electrode pool (Cz, CPz, and Pz) in children. Correlations for the neutral and for the emotional conditions (Table 2) revealed no significant correlations between MMN amplitude and verbal or performance IQ scores in either group. Moreover, between-group comparison of slopes did not reveal any significant difference (Table 2). Overall, these findings suggest that MMN results are not likely to be explained by intellectual discrepancies between groups (Table 1).

**Table 2:** Correlation slopes between electrophysiological measures and verbal or performance IQ scores in the CTRL and ASD children groups and between-group comparisons

	Verbal IQ			Performance IQ		
	CTRL	ASD	Group difference	CTRL	ASD	Group difference
MMN amplitude (Cz, CPz, Pz)						
Neutral	.02 [−.03; .11] *p =* .57	−.01 [−.05; .03] *p =* .55	.03 *p =* .25	−.06 [−.15; .01] *p =* .21	.02 [−.04; .08] *p =* .61	*−*.08 *p =* .09
Emotional	−.01 [−.08; .11] *p =* .84	.00 [−.03; .05] *p =* .76	*−*.01 *p =* .59	−.02 [−.14; .07] *p =* .71	.06 [−.01; .12] *p =* .10	*−*.08 *p =* .03
MMN emotional laterality effect (C4, CP4, P4)	.01 [−.06; .10] *p =* .87	.04 [−.01; .09] *p =* .10	*−*.03 *p =* .26	*−*.01 [−.12; .08] *p =* .88	.04 [−.03; .13] *p =* .35	*−*.05 *p =* .23
MMN latency (Cz)						
Neutral	−.16 [− 1.17; .56] *p =* .61	*−*.07 [−.41; .33] *p =* .70	*−*.09 *p =* .75	−.29 [−.83; .49] *p =* .46	*−*.06 [−.73; .63] *p =* .84	*−*.23 *p =* .49
Emotional	−.08 [−.82; .89] *p =* .80	*−*.03 [−.32; .34] *p =* .87	*−*.05 *p =* .88	−.14 [−.74; .50] *p =* .73	.32 [−.24; 1.29] *p =* .40	*−*.46 *p =* .11
P3a amplitude (Fz, FCz, Cz)						
Neutral	.00 [−.09; .10] *p =* .95	*−*.04 [−.09; .03] *p =* .20	.04 *p =* .27	−.03 [−.14; .10] *p =* .63	*−*.03 [−.16; .10] *p =* .68	−.00 *p =* .96
Emotional	−.03 [−.10; .04] *p =* .48	*−*.07 [−.14; −.00] *p =* .11	.04 *p =* .47	.05 [−.03; .16] *p =* .34	*−*.05 [−.26; .06] *p =* .53	.10 *p =* .49
P3a latency (FCz)						
Neutral	.05 [− 1.24; .88] *p =* .92	*−*.22 [− 1.09; .27] *p =* .42	.27 *p =* .50	.38 [−.84; 1.17] *p =* .48	*−*.61 [− 1.41; .15] *p =* .17	.99 *p =* .04
Emotional	.45 [−.29; .89] *p =* .20	*−*.12 [− 1.07; .35] *p =* .68	.57 *p =* .08	*−*.35 [− 1.15; .62] *p =* .42	*−*.32 [− 1.56; .55] *p =* .52	*−*.03 *p =* .95

All results displayed in black are non-significant (Bonferroni-corrected *p* value threshold: *p* = .0056; nine correlations in each group)

Laterality effect was characterized with an ANOVA performed on C3, CP3, P3, C4, CP4, and P4 in children only in accordance with the results of the main ANOVA. A condition by laterality by group interaction (F(1,28) = 3.87, *p* = .059, *η*_p_^2^ = .12) tended to be significant. This result together with the right-laterality effect of emotional MMN reported in the literature [40] led us to perform a post hoc analysis. A lateralization of the angry MMN to the right hemisphere was evidenced in CTRL children (*p* = .042) while a symmetrical response was observed in children with ASD (*p* = .684, ns). No lateralization was showed for the neutral condition.

Correlation analyses between IQ measures and emotional MMN amplitude performed over the right centro-parietal electrode pool (average of C4, CP4, and P4) did not reveal any significant findings for both within-group correlations and between-group comparisons (Table 2).

#### MMN latency

MMN latency was shorter in participants with ASD compared to CTRL participants (group effect, F(1,58) = 4.39, *p* = .041, *η*_p_^2^ = .07) and also for the angry condition than for the neutral condition (condition effect, F(1,58) = 16.19, *p* < .001, *η*_p_^2^ = .22). MMN latency did not differ between age groups. There were no significant interactions.

No correlation between verbal IQ and MMN latency appeared significant in children or in adults and no between-group comparison *p* value reached the Bonferroni-corrected threshold (Tables 2 and 3). In contrast, the correlation between performance IQ and neutral MMN latency was significant in CTRL adults at the Bonferroni-corrected threshold (Table 3): MMN latency in the CTRL group decreased with increasing performance IQ; therefore, this correlation is not likely to explain the group difference by intellectual discrepancies.

**Table 3:** Correlation slopes between electrophysiological measures and verbal or performance IQ scores in the CTRL and ASD adult groups and between-group comparisons

	Verbal IQ			Performance IQ		
	CTRL	ASD	Group difference	CTRL	ASD	Group difference
MMN amplitude (Cz, CPz, Pz)						
Neutral	*−*.03 [−.06; .01] *p =* .20	.02 [−.05; .08] *p =* .60	*−*.05 *p =* .17	.02 [−.01; .07] *p =* .39	.04 [.00; .08] *p =* .14	*−*.02 *p =* .63
Emotional	−.01 [−.06; .04] *p =* .76	*−*.01 [−.08; .02] *p =* .62	*−*.00 *p =* .91	.04 [−.00; .10] *p =* .19	.00 [−.02; .04] *p =* .80	.04 *p =* .16
MMN latency (Cz)						
Neutral	−.20 [−.67; .30] *p =* .43	.50 [.06; 1.11] *p =* .09	*−*.70 *p =* .04	−.73 [− 1.19; −.19] *p <* .01	*−*.18 [−.66; .22] *p =* .49	*−*.55 *p =* .11
Emotional	−.45 [− 1.45; .47] *p =* .39	*−*.05 [−.49; .09] *p =* .88	*−*.40 *p =* .40	−1.51 [− 2.24; −.82] *p =* .01	*−*.40 [−.89; .04] *p =* .16	−1.11 *p =* .01
P3a amplitude (Fz, FCz, Cz)						
Neutral	*−*.02 [−.06; .03] *p =* .47	.03 [−.04; .08] *p =* .34	*−*.05 *p =* .22	.01 [−.05; .07] *p =* .75	.03 [−.02; .08] *p =* .18	*−*.02 *p =* .65
Emotional	−.02 [−.08; .05] *p =* .67	.00 [−.06; .05] *p =* .94	*−*.02 *p =* .58	.06 [−.01; .15] *p =* .17	.02 [−.03; .07] *p =* .39	.04 *p =* .28
P3a latency (FCz)						
Neutral	.34 [−.42; 1.18] *p =* .40	.18 [−.87; .75] *p =* .62	.16 *p =* .74	*−*.97 [− 2.01; −.04] *p =* .03	*−*.08 [−.62; 0.60] *p =* .78	*−*.89 *p =* .08
Emotional	*−*.39 [−.98; .14] *p =* .19	.11 [−.66; .58] *p =* .68	*−*.50 *p =* .14	.40 [− 1.19; .42] *p =* .27	.03 [−.36; .43] *p =* .89	*−*.43 *p =* .30

All results displayed in black are non-significant (Bonferroni-corrected *p* value threshold *p* = .0063; eight correlations in each group)

Although no interaction between group and condition was observed for MMN latency, the observation of data (Fig. 5c) and the absence of latency difference between neutral and emotional MMN found in children in the literature [45] suggested that the condition effect on MMN latency might be present only in adults. In order to further investigate the existence of this condition effect in our groups, planned comparisons were performed. These analyses revealed that the shorter latency for the emotional condition compared to the neutral condition was significant in both adult groups (CTRL adults *p* = .002; adults with ASD *p* = .007) but not in children groups.

### Orientation of attention to neutral and emotional prosodic changes

#### P3a amplitude

The main ANOVA performed on P3a amplitude revealed a typical fronto-central distribution and a larger P3a for participants with ASD than for CTRL participants (group effect, F(1,58) = 5.44, *p* = .023, *η*_p_^2^ = 0.09). Similar amplitudes were observed between conditions in children while neutral P3a amplitude was larger than angry P3a amplitude in adults (condition by age interaction, F(1,58) = 4.34, *p* = .042, *η*_p_^2^ = 0.07). No other significant result was observed on P3a amplitude.

Finally, P3a amplitude did not correlate with verbal or performance IQ in children or in adults and no between-group differences were observed (Tables 2 and 3).

#### P3a latency

P3a latency was shorter in adults than in children in the ASD group (*p* = .001) but not in the CTRL group regardless of the condition (age by group interaction, F(1,58) = 5.01, *p* = .029, *η*_p_^2^ = 0.08). No other significant result was observed on P3a latency.

For both verbal and performance IQ, no correlations with P3a latency were found in adults or in children and no between-group differences were observed (Tables 2 and 3).

## Discussion

The present study evaluated the early processing of change with vocal stimuli in children and adults with ASD. The goals of this study were to characterize automatic detection of vocal deviancy and to assess whether it is modulated by emotion in people with autism.

An atypical detection of deviancy in vocal stimuli has been evidenced in both adults and children with autism while an absence of specific emotional deviancy response was observed only during childhood.

### Obligatory auditory brain responses in ASD

Before focusing on deviancy processing, auditory brain responses to vocal prosodic stimuli were investigated in an equiprobable context in order to assess voice processing in ASD across age groups. Atypical auditory processing of vocal stimuli was evidenced in children with autism especially for the emotional stimulus. Previous studies investigating voice processing in children with autism [6, 7] did not report any significant difference between groups for vocal stimuli processing. The context of stimulus presentation (speech stimuli only or speech/ non-speech/ non-vocal stimuli) could be responsible for the discrepancy between previous studies and ours as children with autism are sensitive to the stimulus sequence composition [10]. In studies using oddball paradigms composed mostly or even exclusively of speech sounds as in the present study, reductions of ERP amplitude to standard sounds were repeatedly evidenced in children with autism compared to controls [10, 13, 18, 20], including when the stimuli displayed an emotional prosody [40]. Overall, these studies highlight an atypical processing of human voice in children with autism, which could hamper more complex brain processes such as change detection. In adults with autism, no impairment of vocal processing was evidenced in the present work in accordance with a recent fMRI study [9]. Although replication is still necessary to assess the similarity of brain responses to prosody between CTRL and ASD adults, the present findings suggest a normalization of auditory brain responses to vocal sounds with age.

### Atypical deviancy processing for both neutral and emotional conditions in ASD

MMN amplitude tends to normalize according to age but earlier MMN and larger P3a were found in both children and adults with ASD compared to CTRL, highlighting a persistent atypical detection and orientation of attention toward change. The earlier MMN observed in the present study is in contradiction with the few studies that used emotional vocal stimuli in ASD, which either reported no latency difference [40, 41, 62] or a delayed MMN in adults with Asperger syndrome compared to controls [43]. Differences between our findings and those from previous studies might be explained by differences in the paradigm itself or the stimuli. For example, previous studies frequently used a traditional oddball paradigm (in which the differential wave is obtained by subtracting the response to the standard sounds in the oddball sequence from that of the deviants), whereas we used an equiprobable sequence allowing a better control of neural adaptation. Moreover, while previous studies used stimuli like words with complex emotions such as scorn, we presented simple stimuli (vowel) with basic emotions like anger, which might trigger responses that appeared closer to those evoked by simple non-vocal stimuli. Accordingly, our results are consistent with previous investigations using speech stimuli [22] or tones with frequency changes which have already evidenced a faster processing of deviancy in ASD [16, 17]. This earlier response was also reported for non-social stimuli in the visual modality [63]. This would indicate that a general atypical deviancy processing operates in ASD independently of the type (social/non-social) of stimuli and of the sensory modality; this could possibly be related to the need of sameness.

An increased P3a amplitude was found in ASD compared to CTRL contrary to our hypotheses. Though there is limited literature on the P3a response in autism, our result is in contradiction with previous reports [12, 20] including two emotional oddball studies [41, 42], which described smaller P3a in patients with autism. This discrepancy might be explained by the paradigm used (minimizing acoustic differences and neural adaptation effects) or by an instability of involuntary attention across studies varying from low to high awareness. Nonetheless, previous works using tones rather than phonemes have also reported larger P3a responses in participants with ASD in oddball paradigms [16, 17]. Our results add to this by showing a larger P3a response in ASD compared to CTRL for simple vocal stimuli. Larger P3a response indicates a greater involuntary attention to deviancy in ASD which could contribute to the sameness dimension but also suggests that ASD participants noticed emotional changes. The existence of this finding for social vocal stimuli might indicate that this atypical attention orientation to change is a hallmark of the pathology and could be responsible for patients’ difficulties to adapt to their environment [16].

In addition, in children with autism, deviancy processing was also characterized by an MMN amplitude reduction in both conditions compared to CTRL. Amplitude reduction was already evidenced in vocal change detection studies in response to variation in acoustics [11, 18] and emotion [42]. The present finding confirms that this group difference constitutes a general impairment of change detection of vocal stimuli in children with autism.

Contrary to our hypotheses, MMN amplitude reduction was not found for adults with ASD in the present study even though the amplitude tended to be smaller compared to CTRL. In previous emotional MMN studies on which our hypotheses were drawn, an amplitude reduction was reported [41, 43] but this result appeared even for non-vocal counterparts of vocal stimuli [41] confirming the major role of acoustic attributes. As first-order acoustical parameters were controlled in the present study, the absence of significant amplitude reduction appears consistent. However, a lack of group difference might be due to heterogeneity in the adult ASD group. To sum up, in both children and adults with ASD, the response pattern is characterized by an earlier MMN and a larger P3a. Both indicate a heightened pre-attentional processing of change. In children with ASD, despite this greater pre-attentional deviancy processing, change detection was also characterized by a smaller MMN suggesting a reduced fine-grained analysis of the characteristics of the change [64], possibly in relation to the altered sensory processing.

### Atypical processing of emotion-specific deviancy detection in ASD

The comparison of brain responses to neutral and emotional deviancy showed a specificity of the emotional change detection in children which was represented by a right-hemispheric lateralization in the control group that was missing in ASD. This absence of lateralization for emotional deviancy was already evidenced in children with Asperger syndrome [40]. Brain regions involved in emotional processing vary according to stimulus type (e.g., stimuli with/without speech content) and attentional level (e.g., implicit/explicit) [65]. Some fMRI studies have evidenced a right-hemispheric specialization for the processing of emotional prosody in control adults [66–68]. In our study, this lateralization of the emotional MMN did not persist in adults possibly because this right-hemispheric lateralization might be present on larger time windows in adults, which would explain its recording in fMRI studies but not in the time scale of the MMN response.

In CTRL and ASD adults, the emotional MMN displayed an earlier latency compared to the neutral condition. This latency difference may originate from a faster processing of emotional stimuli possibly through a subcortical short route involving the amygdala [69]. It may also reflect a delayed processing of neutral stimuli, due to the presence of emotional stimuli in the sequence [70]. Although this finding was already known in CTRL adults [49, 50], it is the first time that it is also reported for adults with ASD, suggesting that adults with autism are not only able to discriminate between neutral and emotional prosody at a pre-attentive level but also display the appropriate response by prioritizing the emotional deviancy processing. Hence, as we recorded pre-attentional responses, people with ASD do not exclusively base their emotional perception on learned compensatory strategies as previously suggested [36]. Moreover, even if the use of ecological stimuli did not allow to control for all voice parameters (such as sound envelope), the equiprobable paradigm used in the present work reduces the odds of an over-processing of first-order acoustic attributes in ASD to explain this emotional deviancy prioritized processing.

Altogether, the present study highlighted that children with autism did not fully show the specific brain response to emotion revealing an atypical processing of emotional deviancy whereas adults with autism display appropriate emotional deviancy brain responses. The evolution between children and adults with ASD also evidenced a trend toward normalization of vocal processing and automatic detection of emotion with age in ASD. Despite this improvement of brain processes involved in the perception of emotional vocal stimuli, some behavioral studies still show deficits in emotion recognition [71] and prosodic production of adults with autism [72, 73]. Abnormal prosodic perception and production represent a significant obstacle to the social integration of persons with ASD. Therapies helping persons with autism to apprehend emotions [74] therefore seem essential to aid people with autism on the social dimension. Indeed, even if adults with autism are able to pre-attentively detect socially relevant changes, they still display social anhedonia [75], probably because the coping strategies they use in order to fit in and increase connections with others are energy consuming [76]. That is why these interventions need to be administered early to children with ASD to possibly trigger changes of perceptual processes as soon as possible in order to ease the “reading” of the environment and improve the clinical evolution of patients.

### Limitations

Although this study brought innovative results about the perception of vocal stimuli in autism, these processes should be investigated on larger samples to assess the relative influence of IQ and diagnosis even if no influence of cognitive skills was evidenced in our study. Additional experiments with different sequences would also be beneficial to determine the influence of the context on the studied brain processes (e.g., an emotional context compared to the neutral context created by the repeated presentation of the neutral standard in the present study). Behavioral results about emotional recognition tested in laboratory and in real social interactions would also have been helpful to assess the potential link between automatic low-level pre-attentional differences and high-level socio-emotional performances. Finally, longitudinal studies will be of great use to properly evaluate the developmental changes in patients with ASD and to confirm the findings of the present study.

## Conclusions

Detection and orientation of attention toward vocal deviancies remained atypical in adults with autism even if greater group differences were reported in children compared to adults. This long-lasting particularity may be a key element of the ASD symptomology, as an atypical perception of social and non-social changes in the environment prevents people with autism to correctly adapt their reactions and may lead to the sensory overload often reported by individuals with ASD.

In addition to this atypical processing of change, children with ASD exhibited an abnormal sensory encoding of neutral and emotional stimuli along with an atypical pre-attentive discrimination of neutral and emotional deviancies. In adults with ASD, auditory sensory encoding was similar to CTRL adults and both groups discriminated neutral and emotional deviancies. These differences between children and adults with ASD indicate that poor sensory encoding during childhood might have hinder the development of normal automatic change detection. However, normal change detection is not mandatory to discriminate between different emotions as long as sensory encoding is typical as indicated by results obtained in adults with ASD. Overall, the present study evidenced a trend toward normalization of vocal processing and automatic detection of emotion with age in ASD.

## Abbreviations

ADI-R: Autism Diagnostic Interview-Revised
ADOS: Autism Diagnostic Observation Schedule
angryDev: Angry deviant stimulus
AS: Asperger syndrome
ASD: Autism spectrum disorder
CTRL: Controls
DSM-5: Diagnostic and Statistical Manual of Mental Disorders, 5th edition
EEG: Electroencephalography
equiAngry: Equiprobable angry stimulus
equiDisgust: Equiprobable disgust stimulus
equiFear: Equiprobable fear stimulus
equiHappy: Equiprobable happy stimulus
equiSad: Equiprobable sad stimulus
equiSurprise: Equiprobable surprise stimulus
ERP: Event-related potential
fMRI: Functional magnetic resonance imaging
IQ: Intellectual quotient
MMN: Mismatch negativity
neutralDev: Neutral deviant stimulus
neutralStd: Neutral standard stimulus
